# Changes in Corneal Higher-Order Aberrations and Ocular Biometric Measurements after Phacoemulsification Combined with Goniosynechialysis in Primary Angle Closure/Glaucoma Patients

**DOI:** 10.1155/2024/5833543

**Published:** 2024-01-12

**Authors:** Jiali Xia, Siqi Guo, Fei Hu, Liqi Fan, Ling Yu, Jian Ye

**Affiliations:** ^1^Department of Ophthalmology, Daping Hospital, Army Medical Center, Army Medical University, Chongqing 400042, China; ^2^Department of Ophthalmology, The Affiliated Hospital of Southwest Medical University, Luzhou 646000, Sichuan, China

## Abstract

**Purpose:**

To compare corneal higher-order aberrations (HOAs), refractive error, and ocular biological parameters before and after phacoemulsification combined with goniosynechialysis (Phaco-GSL) in primary angle closure/glaucoma (PAC/PACG) patients with different axial lengths (ALs).

**Methods:**

In this prospective study, cataract patients diagnosed with PAC/PACG were categorized into two groups based on their ALs: the short AL group (AL ≤ 22.5 mm) and the normal AL group (22.5 < AL ≤ 24.5 mm). The pre- and postsurgery measurements of intraocular pressure (IOP) and best-corrected visual acuity (BCVA) were conducted at 1 day, 1 week, 1 month, 3 months, 6 months, and 12 months. Additionally, the assessments included corneal HOAs, the number of antiglaucoma medications, visual field parameters, manifest refraction, and other ocular biological parameters before surgery and at the final follow-up.

**Results:**

Prior to surgery, the two groups exhibited no significant differences, except for AL, curvature value, and *Z* (4, 0) of the posterior corneal surface (all *P* < 0.01). Following surgery, BCVA improved, and IOP decreased significantly in both groups (*P* < 0.01). Both anterior and total corneal HOAs, along with *Z* (3, −3), increased in the two groups (all *P* < 0.05), with the normal AL group exhibiting a significantly greater increase in total cornea *Z* (3, −3) than the short AL group (*P*=0.047). The normal AL group also exhibited a slight tendency towards hyperopia (*P* < 0.01). Significant changes were observed in the visual field index and mean deviation in both groups (*P* < 0.05).

**Conclusions:**

Phaco-GSL resulted in an increased corneal HOAs, particularly trefoil, with variations based on the patient's AL. Patients with normal ALs tended to shift towards hyperopia after surgery.

## 1. Introduction

Cataracts and glaucoma are the leading global causes of blindness, with Asians experiencing a higher incidence of primary angle closure (PAC) and PAC glaucoma (PACG) than Europeans and Africans [1]. As the global population ages, there has been an increase in cases of concomitant PAC/PACG and cataract [2]. In recent years, ophthalmologists have shown interest in a surgical technique called phacoemulsification combined with goniosynechialysis (Phaco-GSL), which involves stripping the peripheral anterior synechia (PAS) from the angle wall and restoring trabecular filtering function and has proven effective in lowering intraocular pressure (IOP) and preventing scarring of the filtering bleb after glaucoma filtration surgery [2–4].

Mounting evidence indicates that cataract surgery can induce corneal aberrations due to surgical incisions and alterations in ocular biological parameters, leading to refractive errors and abnormal visual quality [5, 6]. These higher-order aberrations (HOAs), such as trefoil, coma, and spherical aberrations, are significant distortions that cause visual disturbances such as dazzles and starbursts and have been associated with surgical procedures [5–8]. HOAs are optical imperfections of the eye that can affect the retinal image quality and result in visual symptoms. Factors such as age, refractive error, near work, and accommodation can have varying effects on the type and magnitude of HOAs [9]. Furthermore, during cataract development, the number of internal ocular HOAs tends to increase, with notable positive shifts in coma RMS and primary spherical aberrations (Z0,4) in cortical and nuclear cataracts, respectively [10, 11]. The impact of HOAs on visual quality may vary among individuals, depending on factors such as pupil size, amount of aberration, individual visual system, and adaptability. Strategies such as refractive operations or the implementation of appropriate lenses may be considered to mitigate the effects of these aberrations on the visual quality. To the best of our knowledge, only a few studies have investigated corneal HOAs in patients with PAC/PACG and cataracts. Furthermore, PAC/PACG eyes exhibit distinct characteristics, including shallower anterior chambers, shorter axial lengths (ALs), zonular weakness, and larger lens capsule sizes [12], which complicate the prediction of intraocular lens (IOL) positioning after surgery. Determining IOL power in PAC/PACG eyes remains challenging, necessitating surgeons to warn patients about the higher risk of postoperative refractive error. Some studies have found a correlation between AL and unpredictable refractive outcomes after cataract surgery, with a longer AL being associated with greater refractive error [13, 14]. Additionally, the glaucoma filtration surgery typically results in a decreased AL [15]. Therefore, the selection of IOLs for PAC/PACG patients should differ from that for patients with age-related cataracts only.

Given the limited available literature on this subject, our study aimed to compare corneal HOAs, ocular biological parameters, and refractive errors before and after phaco-GSL in patients with different ALs. We sought to identify factors associated with refractive outcomes and compare the success rate of IOP reduction after surgery.

## 2. Patients and Methods

### 2.1. Patient Selection

Consecutive patients scheduled for phaco-GSL at the Daping Hospital of Chongqing, China, between January 2020 and July 2021 were enrolled in this prospective group study. The study and all examinations adhered to the Declaration of Helsinki on Ethical Principles for Medical Research Involving Human Subjects and received approval from the hospital's Ethics Committee (TDLL 2020–90). All participants provided informed consent prior to inclusion in the study.

### 2.2. Inclusion and Exclusion Criteria

This study included patients aged ≥50 years with a diagnosis of PAC/PACG and concomitant cataracts. The diagnosis of PAC or PACG adhered to the criteria outlined by the International Society of Geographic and Epidemiologic Ophthalmology [16]. For PAC patients, the diagnosis necessitated at least 180° of iris trabecular contact during a gonioscopic examination, accompanied by increased IOP or PAS, without a diagnosis of glaucomatous optic neuropathy. In the case of PACG patients, the diagnosis required evidence of glaucomatous optic nerve damage in addition to the aforementioned criteria. The exclusion criteria were fixed dilated pupils, secondary angle closure due to uveitis or ocular trauma, lens subluxation, ocular AL ≤20 mm, and a history of ocular surgery or other ocular diseases. Upon recruitment, patients were categorized into two groups based on their ALs: the short AL group (AL ≤ 22.5 mm) and the normal AL group (22.5 mm < AL ≤ 24.5 mm) [17, 18].

### 2.3. Clinical Data Measurement

Before surgery, a comprehensive ophthalmic examination was performed on all patients. The collected data included patient demographics (age, sex, and number of antiglaucoma medications), IOP, manifest refraction, astigmatism, best-corrected visual acuity (BCVA), AL, keratometry (*K*) values, anterior chamber depth (ACD), corneal HOAs, and visual field parameters. The IOL-Master700 (Carl Zeiss Meditec, Jena, Germany) was used to measure the AL and *K* values, and the online Barrett Universal II formula was used for the IOL calculations. The ACD was measured using ultrasound biomicroscopy (Tianjin Suowei Electronic Technology Co., Ltd.). Visual field parameters were obtained using the Humphrey Field Analyzer 750II (Carl Zeiss Meditec, Dublin, CA). The IOP and BCVA were reassessed on the first postoperative day and during subsequent follow-up visits (1 week, 1 month, 3 months, 6 months, and 12 months). Manifest refraction, ACD, visual field parameters, AL and *K* values, and the number of antiglaucoma medications were recorded again during the final follow-up visit. Combined drops were counted as two medications. Corneal HOAs were measured using a rotating Scheimpflug camera (Pentacam HR, Oculus, Wetzler, Germany) in a dark room, maintaining a 6.0-mm pupillary diameter. The image was selected when the instrument displayed “OK.” The Zernike coefficients of individual patients were analyzed, including the root-mean-square (RMS) of HOAs, primary spherical aberration *Z* (4, 0), horizontal coma *Z* (3, 1), vertical coma *Z* (3, −1), oblique trefoil *Z* (3, 3), and vertical trefoil *Z* (3, −3) of the total cornea, anterior cornea surface, and posterior cornea surface. Multiple measurements were averaged for each parameter.

The success rate of the procedure was determined using the following criteria: complete success, defined as achieving an IOP of 5 mmHg ≤ IOP ≤ 21 mmHg and at least ≥20% reduction in IOP without any glaucoma medications; qualified success, defined as achieving an IOP of 5 mmHg ≤ IOP ≤ 21 mmHg and at least ≥20% reduction in IOP with glaucoma medications; and failure, defined as achieving an IOP >21 mmHg on maximally tolerated medications [19].

### 2.4. Surgical Technique

A single experienced surgeon (LY) performed all Phaco-GSL surgeries. The procedure commenced with topical anesthesia, followed by standard phacoemulsification using a 2.8-mm clear corneal incision at the 10 o'clock position and a 1-mm lateral incision. Subsequently, a hydrophilic acrylic intraocular lens (IOL) (Akreos AO, Bausch & Lomb Inc., Rochester, NY, USA) was implanted into the capsule. The operating microscope was angled at approximately 45°, and the Swan Jacobs direct gonio lens (Ocular Instruments, Bellvue, WA, USA) was placed on the cornea for angle visualization. Viscoelasticity was measured along the peripheral anterior chamber to facilitate the opening of the PAS. A modified iris repository was used to separate the residual PAS and expose the trabecular meshwork. The viscoelastic material was thoroughly removed, and the incision was sealed by corneal stromal hydration. After surgery, all patients received prescriptions for pranoprofen medications (Pranopulin) and tobramycin dexamethasone eye drops (TobraDex) to be applied four times daily, with gradual dose tapering over 4–6 weeks.

### 2.5. Statistical Analysis

All statistical analyses were performed using SPSS software (version 17.0; SPSS Inc., Chicago, IL, USA). Continuous data are expressed as the mean ± standard deviation. The manifest refraction was converted into SEs for the analysis. A paired *t-*test was used to compare pre- and postsurgery parameters. For missing data points that persisted throughout follow-up, the mean completer method was used to fill in the missing values, and the generalized estimating equation was employed to compare follow-up IOP and BCVA at various time points. When comparing data between the two groups, *t*-tests were used for normally distributed data, and the Mann–Whitney *U*-test was used for non-normally distributed data. Pearson's correlation analysis was conducted for normally distributed data, whereas Spearman's correlation analysis was conducted for non-normally distributed data. A *P* value <0.05 was considered statistically significant.

## 3. Results

Initially, 119 eyes from 119 patients were enrolled in the study. However, 23 eyes (12 eyes in the short AL group and 11 eyes in the normal AL group) were subsequently excluded because of failure to meet the inclusion criteria, refusal to participate, or loss of follow-up. Finally, 96 eyes from 96 patients were included in the final analysis, with 48 eyes in the short AL group (mean age: 64.48 ± 6.58 years) and 48 eyes in the normal AL group (mean age: 67.38 ± 8.10 years). No intra- or postoperative complications were observed in either group. The baseline demographic and ocular characteristics of the patients are shown in Table 1. Notably, except for the AL, *K* values, and *Z* (4, 0) of the posterior corneal surface (all *P* < 0.01), no significant differences were noted between the groups, with both groups comprising a higher proportion of female patients.

**Figure 1:**
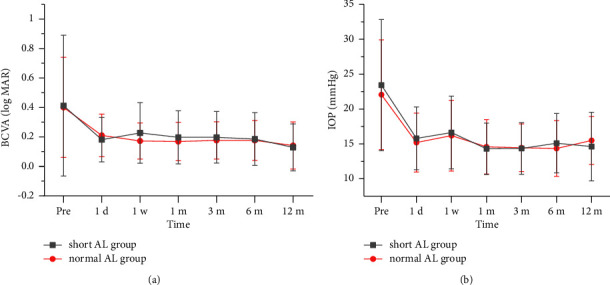
Time-dependent changes in BCVA (LogMAR chart records) and IOP in the two groups. (a) Time-dependent changes in BCVA: the postoperative BCVA was lower than at baseline in both groups at each time point. Error bars represent standard error values. (b) Time-dependent changes in IOP: the postoperative IOP was significantly lower than at baseline in both groups at each time point.

Figure 1 shows the time-dependent changes in BCVA (LogMAR chart records) and IOP. Preoperatively, the mean IOP with drug treatment was 23.42 ± 9.39 mmHg in the short AL group and 22.06 ± 7.89 mmHg in the normal AL group. Generalized estimation equation analysis demonstrated a significant decrease in IOP at each follow-up compared with that at baseline in both groups (*P* < 0.05). The IOP had stabilized at the 1-month follow-up. Similarly, the mean preoperative BCVA was 0.41 ± 0.48 in the short AL group and 0.40 ± 0.34 in the normal AL group. Generalized estimation equation analysis demonstrated a significant improvement in BCVA at each postoperative follow-up compared with that at baseline in both groups (*P* < 0.05). At the final follow-up, BCVA improved to 0.13 ± 0.16 in the short AL group and 0.14 ± 0.16 in the normal AL group. In addition, the number of antiglaucoma drugs decreased from 2.60 ± 1.01 to 0.90 ± 0.88 in the short AL group and from 2.85 ± 0.80 to 0.69 ± 0.85 in the normal AL group, with no significant difference between the groups (*P* > 0.052). The success rate at the final follow-up was 91.67% in the short AL group, comprising complete and qualified success rates of 47.92% and 43.75%, respectively, and a failure rate of 8.33%. The success rate was 95.83% in the normal AL group, with complete and qualified success rates of 52.08% and 43.75%, respectively, and a failure rate of 4.17%.

Table 2 presents changes in corneal HOAs. Before surgery, the short AL group demonstrated significantly higher HOAs and posterior corneal *Z* (4, 0) than the normal AL group (*P*=0.036, *P* < 0.01, respectively). After surgery, the normal AL group showed a significantly greater increase in total cornea *Z* (3, −3) than the short AL group (*P*=0.047). When comparing changes in HOAs pre- and postsurgery within each group, both groups demonstrated an increase in postoperative HOAs and *Z* (3, −3) of the anterior surface and total cornea at the final follow-up (all *P* < 0.05). Moreover, the short AL group demonstrated an increase in anterior surface *Z* (3, 3) and posterior corneal *Z* (4, 0) (*P*=0.009, *P*=0.005). On the other hand, the normal AL group demonstrated an increase in posterior corneal HOAs (*P*=0.021).

Refractive status is categorized as myopia (≤−0.50 D), emmetropia (−0.50 to +0.50 D), and hyperopia (≥0.50 D). Almost half of the patients in each group demonstrated hyperopia, with the short AL group exhibiting a slightly higher proportion (*n* = 23, 47.9% versus *n* = 23, 43.8% in the normal AL group). Emmetropia was present in approximately one-third of the patients in both groups (short AL group: *n* = 18, 37.5% vs. normal AL group: *n* = 14, 29%). Only a small percentage of patients were myopic in the short AL group (14.6%, *n* = 7), whereas the normal AL group had a higher myopic representation (27.1%, *n* = 13). Table 1 shows the refractive error results, defined as the difference between the predicted and actual postoperative SE. No significant differences were observed in the preoperative and predicted SE between the groups (*P*=0.201 and *P*=0.600, respectively); however, a significant difference was found in the postoperative refractive error between the groups (*P*=0.046). The normal AL group displayed a slight shift towards hyperopia (*P* < 0.01), whereas the short AL group did not exhibit a significant difference (*P*=0.756).  Table 3 shows an increased ACD postoperatively in both groups (*P* < 0.01), with no significant difference between the groups (*P*=0.396, *P*=0.233). Compared with the preoperative baseline, there were no significant differences in the K values and AL changes between the groups (*P* > 0.05).

Visual field assessment using the 24-2 SITA standard test indicated no significant differences in the mean deviation (MD), visual field index (VFI), or pattern standard deviation (PSD) between the short and normal AL groups (*P* > 0.05; Table 1). Further examination, as shown in Table 4, revealed a reduction in MD (both *P* < 0.01) and an improvement in VFI (both *P* < 0.01) in both groups after surgery. However, there were no significant changes in the PSD in the either group (*P*=0.05, short AL group; *P*=0.052, normal AL group).

## 4. Discussion

Currently, Currently, Pd data and the Mann-d the study, performed the visualization, wrote the original draft, and wrote, reviewed, and edihaco-GSL is the primary treatment for PACG/PAC with cataracts. However, a discrepancy often exists between the expected and postoperative visual image quality, leading to suboptimal efficacy [20]. A case-control study highlighted that PACG patients may experience a higher incidence of refractive errors after phacoemulsification than those with a single cataract or primary open-angle glaucoma [21]. The presence of a shallow ACD and short AL in PACG patients contributes to poor refractive accuracy [21]. HOAs also affect postoperative visual quality [7, 8]. However, limited research has been conducted on changes in HOAs and ocular biological characteristics in PACG/PAC patients with concomitant cataracts and different ALs. Therefore, in this study, we investigated these changes to identify the influential factors.

We observed an increase in HOAs, particularly *Z* (3, −3) of the anterior surface and total cornea, in both groups at the final follow-up, consistent with the findings of a previous randomized controlled trial conducted by Marcos et al. [22], which demonstrated a significant increase in corneal trefoil and tetrafoil after cataract surgery without any significant changes in spherical aberration or coma. The increase in HOAs could potentially be attributed to corneal inflammation, edema, or remodeling of endothelial cells during the early stages of surgery, which gradually resolves over time [23, 24]. Although further research is needed to fully understand the role of HOAs in visual function, our study revealed, at the same cataract incision size, different increments in the anterior surface and total cornea *Z* (3, −3) in each group, indicating potential differences in visual quality.

Trefoil, classified as an HOA, can result in misfocusing of light on the retina, leading to blurred or distorted vision, reduced contrast sensitivity, compromised depth perception, halos, and starbursts, particularly in environments with low lighting conditions. Previous studies have established a correlation between trefoil and AL, highlighting its strengthening after surgery without compensatory adjustments from the internal optics of the eye [25, 26]. Moreover, a deeper anterior chamber can alter the trajectory of light upon entering the eye, potentially triggering an increase in HOAs. The variation in AL between the groups and alterations in ACD after surgery, as observed in this study, may explain the increase in *Z* (3, −3) after Phaco-GSL. Among the patients included in this study, no significant difference was noted in predicted SE between the two groups. However, the most recent postoperative manifest refraction revealed a tendency for the normal AL group to incline toward hyperopia after surgery.

Our observations also revealed that the ACD deepened in both groups after Phaco-GSL. Cataract removal can induce ACD deepening and subsequently trigger a hyperopic shift in PACG cases where the IOL is implanted in a more posterior position than initially planned [21]. However, the precise mechanism underlying these refractive changes remains unclear. Factors such as large lens capsule size, IOL tilt, or eccentricity can contribute to ineffective lens positioning [27]. Although numerous studies have reported the occurrence of refractive shifts after cataract surgery, the underlying mechanisms remain elusive. Phaco-GSL involves the removal of the lens and deepening of the anterior chamber. According to Ning et al. [27], patients with a shorter preoperative ACD and AL tend to experience greater changes in ACD, leading to a drift towards myopia after cataract surgery. In contrast, patients with a deep preoperative ACD and long AL are more prone to drift towards hyperopia. These findings underscore the predictive value of the ACD in determining refractive outcomes after age-related cataract surgery. Moreover, several studies have highlighted ACD as an indicator of the postoperative position of the IOL, with each 1-mm change in ACD potentially resulting in a refractive shift of at least 0.32D [27, 28].

Visual field examination revealed improvements in MD and VFI after surgery in both groups, with no significant changes in PSD. This suggests that PSD, which represents pathological visual field loss due to lesions, remained unchanged after cataract extraction. These findings were consistent with those of a previous study [29]. Moreover, a reduction in the postoperative use of antiglaucoma medications and high success rates were observed, which are consistent with previous findings [30].

However, it is important to acknowledge the limitations of this study. No PAC/PACG patients with AL >24.5 mm were included during the study period, which is consistent with other studies focusing on the AL of the PAC in Asian populations [31, 32]. Additionally, the absence of a control group undergoing cataract surgery alone among PACG/PAC patients makes it challenging to ascertain whether the observed changes in refractive and corneal HOAs are exclusively associated with GSL separation. Furthermore, although we explored the changes in corneal HOAs among patients, whether these aberrations were associated with vision quality has not yet been elucidated. Further research with larger sample sizes is imperative to investigate the mechanisms underlying changes in corneal HOAs and refractive errors after surgery in PACG/PAC patients with cataracts and different ALs.

## 5. Conclusion

In conclusion, our study findings indicated that phaco-GSL led to increased corneal HOAs, particularly trefoil, at the 1-year follow-up. These changes varied depending on the ALs of the patients. Notably, the change in ACD was found to be the primary influencer of the refractive error. Patients with normal ALs experienced a tendency towards a shift to hyperopia after surgery. Further prospective studies with larger sample sizes are warranted to elucidate the roles of different ALs in ocular changes after phaco-GSL.

## Figures and Tables

**Table 1:** Patients' baseline demographics.

	AL ≤ 22.5 mm	22.5 < AL ≤ 24.5 mm	*P*
Eyes (*n*)	48	48	—
Male/female	4/44	16/32	0.003
Mean age (y)	64.48 ± 6.58	67.38 ± 8.10	0.058
Nuclear grade of cataract	2–4	2–4	—
Mean AL (mm)	21.82 ± 0.53	23.26 ± 0.52	<0.01
*K* value (D)	45.76 ± 1.46	43.49 ± 0.98	<0.01
Astigmatism (D)	−0.77 ± 0,65	−0.64 ± 0.40	0.228
ACD (mm)	2.04 ± 0.28	2.09 ± 0.37	0.536
SE (D)	0.52 ± 1.65	−0.08 ± 2.18	0.201
IOP (mmHg)	23.42 ± 9.39	22.06 ± 7.89	0.673
BCVA	0.41 ± 0.48	0.40 ± 0.34	0.251
Predicted SE (D)	−0.27 ± 0.28	−0.38 ± 0.37	0.600
Number of antiglaucoma medications	2.60 ± 1.01	2.85 ± 0.80	0.238

*P* < 0.05 was considered statistically significant. ACD: anterior chamber depth; AL: axial length; D: diopter; IOP: intraocular pressure; SE: spherical equivalent; BCVA: best-corrected visual acuity.

**Table 2:** Comparison of changes in corneal HOAs between the two groups before and after surgery.

Corneal HOAs	AL ≤ 22.5 mm		22.5 < AL ≤ 24.5 mm	
	Before operation	After operation	Before operation	After operation
*Anterior corneal surface*				
HOAs	0.64 ± 0.35	0.77 ± 0.40^†^	0.63 ± 0.41	0.77 ± 0.34^†^
*Z* (4, 0)	0.32 ± 0.14	0.36 ± 0.17	0.30 ± 0.16	0.31 ± 0.19
*Z* (3, −1)	0.08 ± 0.37	0.15 ± 0.39	0.10 ± 0.27	0.12 ± 0.28
*Z* (3, 1)	0.04 ± 0.20	0.07 ± 0.22	0.00 ± 0.17	0.03 ± 0.19
*Z* (3, 3)	−0.05 ± 0.17	−0.10 ± 0.24^†^	−0.04 ± 0.19	−0.12 ± 0.22
*Z* (3, −3)	−0.02 ± 0.19	−0.22 ± 0.22^†^	−0.13 ± 0.21	−0.36 ± 0.26^†^

*Posterior corneal surface*				
HOAs	0.21 ± 0.06	0.23 ± 0.08	0.19 ± 0.08^*∗*^	0.21 ± 0.07^†^
*Z* (4, 0)	−0.13 ± 0.04	−0.15 ± 0.04^†^	−0.09 ± 0.04^*∗*^	−0.10 ± 0.05
*Z* (3, −1)	0.01 ± 0.05	−0.00 ± 0.04	0.04 ± 0.06	0.03 ± 0.06
*Z* (3, 1)	−0.00 ± 0.05	0.01 ± 0.05	0.00 ± 0.06	0.01 ± 0.05
*Z* (3, 3)	0.01 ± 0.06	0.00 ± 0.07	0.01 ± 0.08	−0.01 ± 0.06
*Z* (3, −3)	−0.03 ± 0.07	0.02 ± 0.08	−0.03 ± 0.06	−0.00 ± 0.07

*Total cornea*				
HOAs	0.61 ± 0.36	0.73 ± 0.43^†^	0.62 ± 0.32	0.79 ± 0.36^†^
*Z* (4, 0)	0.29 ± 0.13	0.33 ± 0.18	0.30 ± 0.17	0.30 ± 0.20
*Z* (3, −1)	0.11 ± 0.40	0.15 ± 0.41	0.15 ± 0.29	0.14 ± 0.30
*Z* (3, 1)	0.04 ± 0.19	0.07 ± 0.22	0.00 ± 0.18	0.04 ± 0.20
*Z* (3, 3)	−0.05 ± 0.18	−0.10 ± 0.21	−0.05 ± 0.18	−0.13 ± 0.21
*Z* (3, −3)	−0.05 ± 0.19	−0.21 ± 0.22^†^	−0.15 ± 0.21	−0.37 ± 0.27^§†^

^*∗*^: *P* < 0.05, significant differences between the two groups before surgery. ^†^: *P* < 0.05, compared with the preoperative baseline in each group. ^§^: *P* < 0.05, significant differences between two groups after surgery. AL: axial length; HOAs: higher-order aberrations; *Z* (4, 0): spherical; *Z* (3, 1): horizontal coma; *Z* (3, −1): vertical coma; *Z* (3, 3): horizontal trefoil; *Z* (3, −3): vertical trefoil.

**Table 3:** Comparison of ocular biological parameters between the two groups.

Parameters	AL ≤ 22.5 mm		22.5 < AL ≤ 24.5 mm	
	Before operation	After operation	Before operation	After operation
AL (mm)	21.82 ± 0.53	21.73 ± 0.53	23.26 ± 0.52^*∗*^	23.14 ± 0.50
ACD (mm)	2.04 ± 0.28	3.72 ± 0.39^†^	2.09 ± 0.37	3.91 ± 0.39^†^
*K* value (D)	45.76 ± 1.46	45.72 ± 1.47	43.49 ± 0.98^*∗*^	43.42 ± 0.98

^*∗*^: *P* < 0.05, significant differences between the two groups before surgery. ^†^: *P* < 0.05, compared with the preoperative baseline in each group. ACD: anterior chamber depth; AL: axial length; D: diopter.

**Table 4:** Comparison of visual field parameters between the two groups.

Parameters	AL ≤ 22.5 mm		22.5 < AL ≤ 24.5 mm	
	Before operation	After operation	Before operation	After operation
VFI	86.57 ± 17.00	90.29 ± 14.54^†^	85.06 ± 19.53	89.13 ± 13.51^†^
MD	−6.95 ± 6.23	−4.78 ± 5.48^†^	−6.94 ± 5.95	−5.33 ± 4.53^†^
PSD	4.00 ± 3.52	3.73 ± 3.82	4.34 ± 3.40	4.06 ± 3.59

^*∗*^: *P* < 0.05, significant differences between the two groups before surgery. ^†^: *P* < 0.05, compared with the preoperative baseline in each group. AL: axial length; VFI: visual field index; MD: mean deviation; PSD: pattern standard deviation.

## Data Availability

The data used to support the findings of this study are available from the corresponding author upon request.
